# Bioequivalence Study of Two Long-Acting Formulations of Oxytetracycline Following Intramuscular Administration in Bovines

**DOI:** 10.3389/fvets.2016.00050

**Published:** 2016-06-23

**Authors:** Nora Mestorino, María Laura Marchetti, Mariana Florencia Lucas, Pilar Modamio, Pedro Zeinsteger, Cecilia Fernández Lastra, Ignacio Segarra, Eduardo Luis Mariño

**Affiliations:** ^1^Laboratory of Pharmacological and Toxicological Studies (LEFyT), Faculty of Veterinary Science, Universidad Nacional de La Plata, La Plata, Argentina; ^2^Clinical Pharmacy and Pharmacotherapy Unit, Department of Pharmacy and Pharmaceutical Technology, Faculty of Pharmacy, University of Barcelona, Barcelona, Spain

**Keywords:** oxytetracycline, pharmacokinetics, AUC, *C*_max_, *T*_max_, bioequivalence, bovines

## Abstract

The aim of this study was to evaluate the bioequivalence of two commercial long-acting formulations based on oxytetracycline (OTC) hydrochloride between the reference formulation (Terramycin LA, Pfizer) and a test formulation (Cyamicin LA, Fort Dodge Saude Animal). Both formulations were administered in a single intramuscular route at a dose of 20 mg OTC/kg of body weight in clinically healthy bovines. The study was carried out according to a one-period parallel design. Plasma samples were analyzed by high-pressure liquid chromatography. The limit of quantitation was 0.050 μg/mL with an accuracy of 101.67% with a coefficient of variation of 13.15%. Analysis of variance and 90% confidence interval tests were used to compare the bioavailability parameters (maximum plasma concentration, *C*_max_, and the area under the concentration-versus-time curve extrapolated to infinity, AUC_0–∞_) of both products. In the case of the time to maximum concentration (*T*_max_), non-parametric tests based on Wilcoxon’s signed rank test were preferred. The comparison of the mean AUC_0–∞_ values did not reveal any significant differences (311.40 ± 93.05 μg h/mL and 287.71 ± 45.31 μg h/mL, respectively). The results were similar for the *T*_max_ (3.58 ± 0.90 h versus 3.42 ± 0.51 h). However, when comparing the mean *C*_max_ some significant differences were found (8.73 ± 3.66 μg/mL and 10.43 ± 3.84 μg/mL, respectively). The 90% confidence intervals for the ratio of AUC_0–∞_ and *T*_max_ values for the reference and test product are within the interval 80–125%, but the 90% confidence intervals for the ratio of *C*_max_ falls outside the proposed interval. It was concluded that *C*_max_ of test product are not within the 20% of those of the reference, thus suggesting that test OTC is not bioequivalent to the reference formulation.

## Introduction

The determination of the bioequivalence of veterinary drug formulations has become an increasingly important issue for both the European Union and the United States of America. Bioequivalence determination guidelines have been established by authorities of both regulatory bodies. For the American guideline, “two products are considered to be bioequivalent when they are equally bioavailable; that is, equal in the rate and extent to which the active ingredients(s) or therapeutic ingredient(s) is (are) absorbed and become(s) available at the site(s) of drug action” (1, 2). European guideline affirms that “bioequivalence exists between veterinary medicinal products or between routes of administration if, under identical and appropriate experimental conditions, the bioavailability of the active ingredient differs within acceptable limits” (3). In other words, both definitions state that equivalent rate and extent of absorption will lead to same plasma drug concentration–time profiles and, therefore, essentially the same magnitude of therapeutic or toxic effects.

The goal of the bioequivalence trial is to demonstrate that, with controlled risk to the patient, two formulations are bioequivalent and, therefore, the practitioner may use them interchangeably.

The determination of drug product bioequivalence is based on a statistical comparison of selected pharmacokinetic parameters of the two formulations of the same drug where the differences in rate and extent of drug absorption are shown to be no more than ±20% with a 90% degree of confidence.

“*In vivo*” experiments are the best way to compare the bioavailability of formulations with theoretically similar effects. In general terms, two formulations are compared, the new formulation (test formulation) versus a reference formulation efficacy of which has been clinically determined. According to the guidelines of the EMEA (3), the pharmacokinetic parameters compared between reference and test formulation in bioequivalence studies after a single dose are the area under the plasma concentration time (Cpt) curve to last concentration (AUC_0–t_) or the area under the Cpt curve extrapolate to infinity (AUC_0–∞_), the peak maximum plasma concentration (*C*_max_), and the time to maximum concentration (*T*_max_). Non-compartmental analysis methods should be used for determination of the pharmacokinetic parameters in bioequivalence studies. If these three parameters deviate within the acceptance range, then the test formulation is considered to be bioequivalent to the reference product.

Briefly, the width of the confidence interval is determined by the within subject variance (between subject variance for parallel group studies) and the number of subjects in the study. In general, the confidence interval for untransformed data should be 80–120% (the confidence interval should lie within ±20% of the mean of the reference product). For logarithmically transformed data, the confidence interval is generally 80–125%.

In veterinary medicine, oxytetracycline (OTC) as well as the other major tetracyclines, are widely used mainly to treat respiratory, gastrointestinal, skin, locomotive organs, and genito-urinary bacterial infections, as well as systemic infections and sepsis (4).

Oxytetracycline is one of the cheapest classes of tetracycline antibiotics available, and this is due to modern manufacturing processes. Such conditions make it particularly attractive for its use in developing countries. The molecule has been available for veterinary medicine for the last half-century.

Due to the difficulties that daily OTC injections represent, long-acting (LA) alternatives were developed to achieve fast and high blood levels and to provide greater effective plasma concentrations during several days (5–7). An ideal OTC formulation for cattle should have some characteristics, such as the ability to maintain antimicrobial concentrations in tissues above the bacterial minimum inhibitory concentration for a long period of time, be easy to administrate, and with minimal tissue irritation or damage. Consequently, parenteral LA injections are used as an alternative for the therapy of diseases that normally require several daily parenteral treatments to provide sustained concentrations of the antibiotic at the site of infection. The formulation provides prolonged circulating antibacterial concentration of the active agent, without the profile produced by repeated injections that may lead to the concentration of the agent in the blood and tissue falling below effective values. Such preparations have been particularly popular in cattle and swine because of the convenience of a single injection (4, 5, 8). Licensed 20 and 30% formulations of OTC have persistent actions because of the high strength and high dosage used (20 or 30 mg/kg), leading to sustained absorption from the reservoir site at the intramuscular injection.

Many OTC pharmacokinetic studies have been conducted in cattle. For generic medicinal products, the purpose of establishing bioequivalence is to demonstrate equivalence in pharmacokinetic parameters between the generic medicinal product (test product) and a reference one (3). Consequently, bioequivalence studies are important for the development of new pharmaceutical formulations (9).

The purpose of this study was to demonstrate the bioequivalence of two LA commercial OTCs, a test formulation (Cyamicin 20% LA) and the reference formulation (Terramycin 20% LA) after intramuscular administration to bovine.

## Materials and Methods

### Chemicals and Reagents

Oxytetracycline hydrochloride of high purity (>95%) was purchased from Sigma Aldrich Chemicals and high-performance liquid chromatography (HPLC) grade solvents were obtained from J.T. Baker®.

### Drugs

Two commercial products of OTC, containing 200 mg of OTC hydrochloride were compared. A reference formulation (Terramycin 20% LA, Pfizer) and a test formulation (Cyamicin 20% LA, Fort Dodge Saude Animal) were used. In order to confirm OTC concentrations, the two formulations were previously analyzed.

### Animals

Twenty four young castrated male Aberdeen Angus bovines weighing 150–280 kg and aged between 6 and 12 months were used in this study. Animals were clinically evaluated to assure satisfactory health status prior to the beginning of the study and were identified with plastic numbered ear-tags.

### Experimental Design

Animals were divided into two groups (group A and group B) of 12 bovines each, according VICH guidelines (VICH GL 52 – Bioequivalence, 2015) (10). The animals were ranked by weight and allocated by Greek guard to one of the two groups, after random allocation of the first animal. The scale was a digital Ezy-weight, made in New Zealand.

Dosage way and route of administration were done following the label instructions of each antibiotic. Only one treatment was carried out per bovine; thus, the study was designed as a one-period parallel study. Parallel designs do not need a washout period between treatments and they are often used for bioequivalence studies conducted in patients or for drugs with a long half-life where crossover studies are difficult or impossible to perform. If the bioequivalence study is problematic, single-dose parallel designs can be an alternative choice, because it is easy to organize, easy to analyze, and easy to interpret. The intramuscular (i.m.) injection was applied on the gluteal area.

– Animals of group A were given a single i.m. dose of 20 mg/kg of the reference (R) product (Terramycin LA, Pfizer).
– Animals of group B received the test (T) formulation (Cyamicin LA, Fort Dodge) at the same dose and by the same route.

The 24 animals were housed in a grass paddock. Feeding was on natural pasture and water was available *ad libitum*. The protocol followed the “Guide for the Care and Use of Agricultural Animals in Agricultural Research and Teaching” (Federation of Animal Science Societies, FASS) and was approved by the Experimental Ethics Committee of the Faculty of Veterinary Science, UNLP, Argentina.

Animals were sampled during the following 5 days after the OTC application. Blood samples were obtained from each animal from the jugular vein at the following post-treatment hours: 1, 2, 3, 4, 6, 8, 10, 12, 15, 18, 24, 36, 48, 60, 72, 96, and 120 to measure the level of active ingredient. Bleeding was done with 10 mL plastic disposal heparinized syringes and 21Gx11/2″ gage. Plasma was obtained by centrifugation and each tube was properly identified and was stored at −20°C until assayed.

Oxytetracycline was assayed by HPLC. Pharmacokinetics and comparative bioavailability were determined.

### Oxytetracycline Assay

Plasma samples were analyzed for OTC determination following an analytical method validated at LEFyT (SOP: OTC-PL-VMA-12/05).

Oxytetracycline was assayed by HPLC with UV detection. For this, 0.1 mL plasma samples were deproteinized by adding 0.2 mL of acetonitrile and vigorously mixing for 5 min. After centrifugation of the samples for 10 min, the supernatant was evaporated to dryness at 45°C under nitrogen stream. The residue was reconstituted with 100% of mobile phase and then a 50 μL aliquot was injected directly into the HPLC system.

The chromatographic system consisted of an isocratic pump (Gilson Inc. 307), an automatic injector (Gilson Inc. 234) and a UV-VIS detector (Gilson Inc. 155) set at a wavelength of 254 nm and an octadecylsilane column (Luna C18, 4.6 mm × 150 mm, 5 μm; Phenomenex, Torrance, CA, USA). The sample was eluted with a mixture of 0.05 M phosphate buffer (pH 4.5) and acetonitrile (85:15, v/v) at a flow rate of 1.5 mL/min and at room temperature. The calibration curve from the standard samples was linear over the concentration range of 0.05–10 μg/mL.

### Precision of the System

One standard solution containing 1 μg/mL of OTC was prepared and the precision of the system was evaluated after the placement of 20 injections in the chromatographic system. Thereby, the efficiencies of the column and of the system were evaluated. After 20 injections a coefficient of variation (CV) of 8.59% was determined.

### Calibration and Validation

Assay was validated by measuring concentrations of known amounts of OTC in plasma of cattle. Linearity, precision, accuracy, recovery, and specificity were determined (*n* = 6).

#### Linearity of Standard Curves

The ratio between different concentrations was determined. Calibration curve was obtained for OTC (concentrations ranging between 0.05 and 10 μg/mL).

#### Precision and Accuracy

The inter-day precision was determined to estimate the run-to-run extraction and chromatographic variation in the method. Inter-day variation was measured during three (3) consecutive working days for plasma samples. Precision was expressed as %CV and accuracy as %ER (11, 12).

Accuracy is defined as the extent to which the experimental values agree with the true values. Accuracy of the method was determined by the differences between desired and calculated concentration results divided by desired concentration, and expressed as the relative error (% RE) (11, 12).

#### Lower Limit of Quantitation

The lower limit of quantitation (LLOQ) was calculated (*n* = 12) as the lowest drug concentration on the standard curve that could be quantitated with CV and relative error not exceeding 20%, and recovery between 20 and 80% of nominal value (11, 12).

### Pharmacokinetic and Statistical Analysis

Pharmacokinetic analyses were performed using WinNonlin Professional version 6.4 (Pharsight Corp.) software. The linear trapezoidal rule was used to calculate the AUC_0–last._ In addition, the elimination rate constant (k_el_) determined from the terminal slope by log-linear regression, T_1/2_, and AUC_0–∞_ calculated as the addition of AUC_0–last_ and C_last_/k_el_, were determined for the analysis.

The one-way analysis of variance (ANOVA) for parallel design was used to assess the effect of formulations on the raw (untransformed) and logarithmically transformed data of AUC_0–∞_, *T*_max_ and *C*_max_ according VICH GL52 (Bioequivalence, August 2015) (10). In the case of *T*_max_, non-parametric tests based on Wilcoxon’s signed rank test were preferred (13). Parametric 90% confidence intervals based on the ANOVA of the mean Test/Reference ratios of AUC_0–∞_, and *C*_max_ were computed under the assumption of multiplicative model using log-transformed data. Confidence intervals were determined by the method of Westlake (14).

## Results

### Assay Linearity

**Figure 1:**
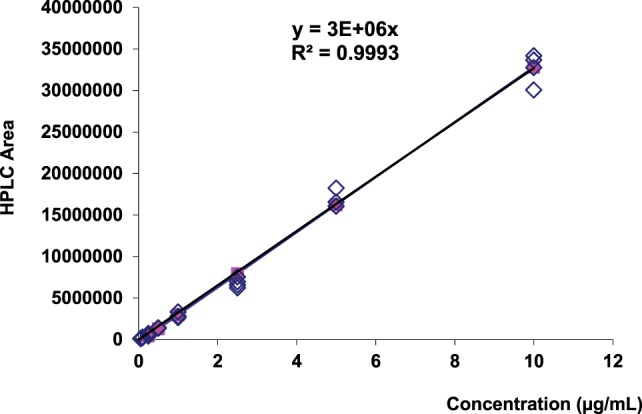
**Calibration curve corresponding to OTC standard**.

This assay exhibited a linear dynamic range between 0.05 and 10 μg/mL with *r*^2^ value >0.999. A linear relationship was obtained across one dynamic range, as determined for the plasma spiked curves shown in Figure 1.

### Limit of Detection

The limit of detection (LOD) is the smallest detectable but not quantifiable quantity of analyte. It was estimated by means of the analysis of 20 aliquots of control plasma (free of antibiotic). The noise of the base-line was measured; the average and the SD were calculated. The LOD corresponds to three of those SDs, which in this case allowed to detect levels of 0.013 μg/mL with a recovery of >85%.

### Intra-Day and Inter-Day Accuracy and Precision

To assess the inter-day (over 3 days) assay accuracy and precision, three sets of plasma samples were spiked with OTC at 0.05, 0.25, 5, and 10 μg/mL concentrations.

To determine the intra-day accuracy and precision, six replicates at each four concentrations were analyzed along with duplicate standard calibration curves prepared from two separate stock solutions.

The intra-day and inter-day variation recovery, precision, and accuracy of the method are illustrated in Tables 1 and 2, respectively. The mean recovery was within the range of 85–115%, and precision and accuracy for the 0.25, 5.00, and 10.00 μg/mL were, respectively, ≤15 and ±15%.

**Table 1:** **Intra-day recovery, precision, and accuracy**.

Desired (μg/mL)	Calculated (μg/mL)	Recovery (%)	Mean	SD	Precision (% CV)	Accuracy (% RE)
0.05	0.05	100.00				
0.05	0.04	80.00				
0.05	0.06	120.00				
0.05	0.05	100.00				
0.05	0.05	100.00				
0.05	0.05	100.00	100.00	12.65	12.65	0.00

0.25	0.2	80.00				
0.25	0.26	104.00				
0.25	0.2	80.00				
0.25	0.22	88.00				
0.25	0.2	80.00				
0.25	0.26	104.00	89.33	11.78	13.18	−12.00

5	4.14	82.80				
5	4.12	82.40				
5	5.13	102.60				
5	4.22	84.40				
5	5.08	101.60				
5	3.82	76.40	88.37	10.98	12.43	−11.60

10	8.26	82.60				
10	8.54	85.40				
10	7.41	74.10				
10	8.47	84.70				
10	10.93	109.30				
10	8.91	89.10	87.53	11.78	13.46	−12.5

*% CV = (SD/calculated concentration) × 100; % RE = (calculated concentration − desired concentration)/(desired concentration × 100)*.

**Table 2:** **Inter-day recovery, precision, and accuracy**.

Desired (μg/mL)	Day 1 (calculate)	% Recovery	Day 2 (calculate)	% Recovery	Day 3 (calculate)	% Recovery	Mean (% Recovery)	SD (% Recovery)	%CV (% Recovery)	Accuracy (%RE)
0.05	0.05	100.00	0.06	120.00	0.04	84.00	101.33	18.04	17.80	
0.05	0.04	80.00	0.05	100.00	0.06	116.00	98.67	18.04	18.28	
0.05	0.06	120.00	0.05	100.00	0.06	120.00	113.33	11.55	10.19	
0.05	0.05	100.00	0.05	100.00	0.06	120.00	106.67	11.55	10.83	
0.05	0.05	100.00	0.06	120.00	0.06	120.00	113.33	11.55	10.19	
0.05	0.05	100.00	0.04	80.00	0.04	80.00	86.67	11.55	13.32	
Mean	0.05	100.00	0.05	103.33	0.05	106.67	103.33			
SD	0.01	12.65	0.01	15.06	0.01	19.21	10.15			
CV%	12.65	12.65	14.57	14.57	18.01	18.01	9.82			0.00

0.25	0.20	80.00	0.23	92.00	0.28	112.00	94.67	16.17	17.08	
0.25	0.26	104.00	0.23	92.00	0.24	96.00	97.33	6.11	6.28	
0.25	0.20	80.00	0.22	88.00	0.24	96.00	88.00	8.00	9.09	
0.25	0.22	88.00	0.21	84.00	0.25	100.00	90.67	8.33	9.18	
0.25	0.20	80.00	0.23	92.00	0.23	92.00	88.00	6.93	7.87	
0.25	0.26	104.00	0.24	96.00	0.24	96.00	98.67	4.62	4.68	
Mean	0.22	89.33	0.23	90.67	0.25	98.67	92.89			
SD	0.03	11.78	0.01	4.13	0.02	7.00	4.67			
CV%	13.18	13.18	4.56	4.56	7.10	7.10	5.03			−8.00

5	4.14	82.80	4.72	94.40	4.18	83.6	86.93	6.48	7.45	
5	4.12	82.40	4.21	84.20	3.98	79.6	82.07	2.32	2.82	
5	5.13	102.60	5.08	101.60	4.01	80.20	94.80	12.65	13.35	
5	4.22	84.40	4.87	97.40	4.87	97.40	93.07	7.51	8.06	
5	5.08	101.60	3.95	79.00	3.98	79.60	86.73	12.88	14.85	
5	3.82	76.40	4.14	82.80	4.21	84.20	81.13	4.16	5.13	
Mean	4.42	88.37	4.50	89.90	4.21	84.10	87.46			
SD	0.55	10.98	0.46	9.11	0.34	6.82	5.57			
CV%	12.43	12.43	10.14	10.14	8.11	8.11	6.37			−12.40

10	8.26	82.60	8.82	88.20	8.09	80.90	83.90	3.82	4.55	
10	8.54	85.40	8.78	87.80	8.99	89.90	87.70	2.25	2.57	
10	7.41	74.10	10.93	109.30	8.77	87.70	90.37	17.75	19.64	
10	8.47	84.70	10.37	103.70	8.93	89.30	92.57	9.91	10.71	
10	10.93	109.30	9.23	92.30	9.21	92.10	97.90	9.87	10.08	
10	8.91	89.10	8.97	89.70	8.94	89.40	89.40	0.30	0.34	
Mean	8.75	87.53	9.52	95.17	8.82	88.22	90.31			
SD	1.18	11.78	0.91	9.09	0.39	3.85	4.72			
CV%	13.46	13.46	9.56	9.56	4.37	4.37	5.23			−9.70

*% CV = (SD/calculated concentration) × 100; % RE = (calculated concentration − desired concentration)/(desired concentration × 100)*.

### Specificity

**Figure 2:**
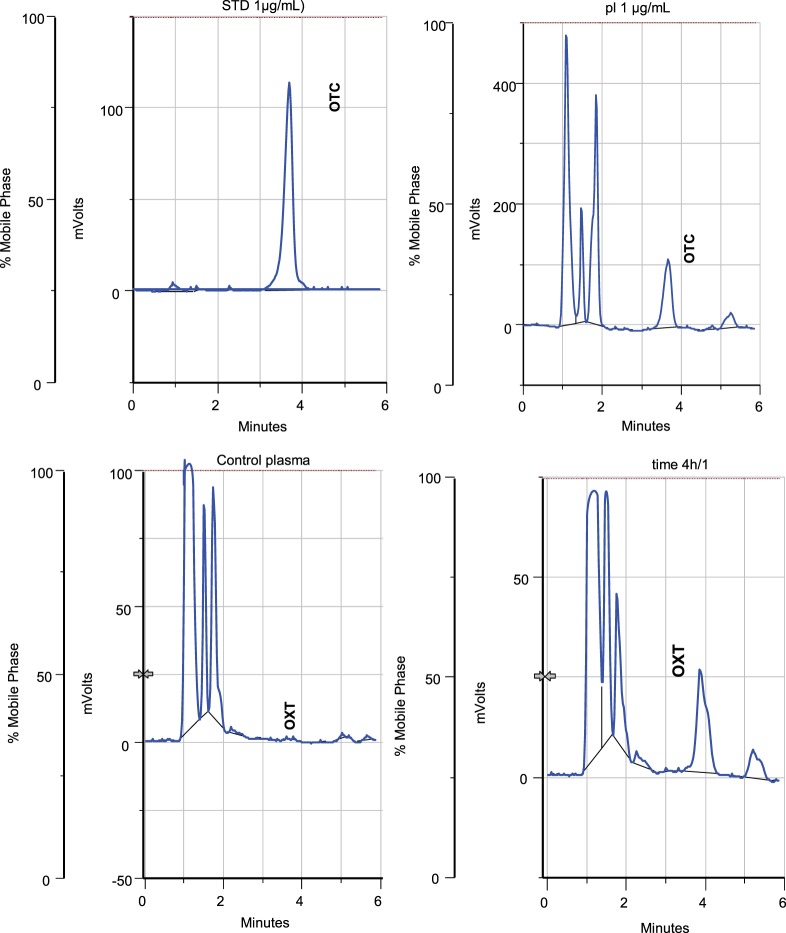
**HPLC chromatograms (UV detector set at 254 nm)**. **(A)** Chromatogram of standard OTC solution (1 μg/mL); **(B)** chromatogram of control plasma sample (free of OTC) after extraction procedure; **(C)** chromatogram after extraction of plasma spiked with OTC (1 μg/mL); and **(D)** chromatogram after extraction of problem sample (animal 1 after 4 h OTC formulation administration).

Six different samples from control plasma (free of antibiotics) and six plasma samples loaded with OTC were analyzed by HPLC and the corresponding chromatograms were compared. With this technique, no interferences in the retention time (3.85 ± 0.14 min) for OTC were found (Figure 2).

### Lower Limit of Quantitation

The lower limit of quantitation was 0.050 μg/mL with a recovery of 103.33%, and a precision and accuracy, respectively, ≤20 and ±20%.

### Pharmacokinetic Analysis

**Figure 3:**
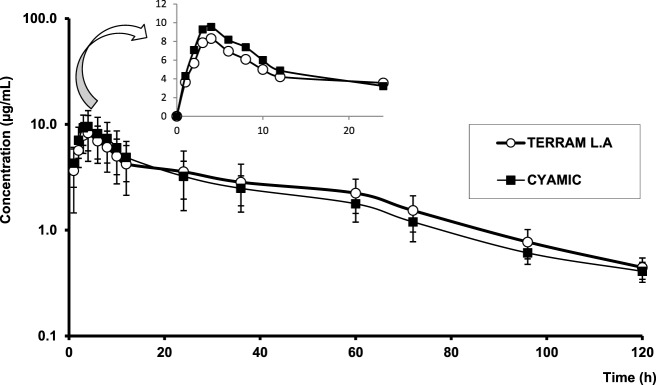
**Semilogarithmic comparative curves (mean ± SD) of oxytetracycline plasma profiles obtained following intramuscular administration of both formulations to bovines**. Small inserted figure: arithmetic comparative curves (0–24 h after each administration).

The mean ± SD OTC plasma concentration–time profiles after the intramuscular administration of each formulation (R and T) to bovines are presented in Figure 3, whereas the intramuscular pharmacokinetic data for Terramycin LA and Cyamicin LA, are presented in Table 3.

**Table 3:** **Comparison of the pharmacokinetic parameters (mean ± SD) of the reference (Terramycin LA) and test (Cyamicin LA) formulations at a single dose (20 mg/kg) administration to bovines**.

		Terramycin		Cyamicin	
Parameter	Unit	Mean	SD	Mean	SD
λ	h^−1^	0.020	0.005	0.022	0.004
T½λ	h	36.88	11.50	32.61	5.14
*C*_max_	μg/mL	8.73	3.66	10.43	3.84
*T*_max_	h	3.58	0.90	3.42	0.51
AUC_0–∞_	μg h/mL	311.40	93.05	287.71	45.31
AUC_0–24_	μg h/mL	118.29	45.46	131.15	44.56

*λ, rate constant associated with the terminal elimination phase; T½λ, elimination half-life; C_max_, maximum concentration; T_max_, time to reach maximum concentration; AUC_0–∞_, area under the concentration–time curve from 0 to infinity; AUC_0–24_, area under the concentration time curve from 0 to 24 h*.

Oxytetracycline plasma concentrations evolved following known features for 20% formulations. Maximum plasma concentrations (*C*_max_) of 8.73 ± 3.66 and 10.43 ± 3.84 μg/mL were obtained for R and T formulations, respectively. Both formulations reached the *C*_max_ between 3 to 4 h. The terminal elimination half-lives (T½λ) of OTC were 36.88 ± 11.50 h and 32.61 ± 5.14 h after administration of Terramycin LA and Cyamicin LA, respectively (Table 3).

### Bioequivalence Analysis

The bioavailability of the Test product (Cyamicin L.A) relative to the Reference product (Terramycin L.A) was compared on the basis of the Log-transformed and untransformed study data. Differences between the R and T products were statistically evaluated by means of confidence intervals. *C*_max_, *T*_max_, and AUC_0–∞_ were analyzed by ANOVA. The criterion for product bioequivalence requires for *C*_max_, *T*_max_, and AUC_0–∞_ that the 90% confidence intervals about the difference in product means (test minus reference values) be within ±20% of the reference mean.

Tables 4 and 5 displays the statistic ANOVA comparison for log-transformation and untransformed data and the Westlake’s 90% CI interval, respectively. Using this criterion, the test product did not demonstrate bioequivalence with the reference product. The parameters *T*_max_ and AUC_0–∞_ of the test formulation were not significantly different from the reference formulation. However, in the case of *C*_max_, T product presented a significantly higher value compared to R, which exceeded the bioequivalence criterion.

**Table 4:** **Statistical comparison between the pharmacokinetic parameters obtained for Terramycin L.A (R) and Cyamicin (T) by ANOVA Test with log transformed and untransformed data**.

Parameter	Reference	Test	ANOVA	ANOVA (log)
AUC	311.40 ± 93.05	287.71 ± 45.31	>0.30	>0.30
*C*_max_	8.73 ± 3.66	10.43 ± 3.84	0.22	0.21
*T*_max_*	3.58 ± 0.90	3.42 ± 0.51	>0.30	>0.30

T_max_*: bioequivalence evaluation by Steinijans and Diletti’s non-parametric test. Arithmetic mean of individual differences = −1.66667E−01.

*90% confidence interval (Test vs. Ref.) = 86.0–114.0% vs. mean Ref*.

**Table 5:** **Westlake’s 90% interval calculated iteratively for log untransformed and transformed data**.

Parameter	Reference	Test	Westlake, interval for untransformed 90% (USA)	Westlake, interval after ln-transformation 90% (log) (CEE)
AUC	311.40 ± 93.05	287.71 ± 45.31	80.58–119.42%	85.89–114.11%
*C*_max_	8.73 ± 3.66	10.43 ± 3.84	60.13–139.87%	65.04–134.97%
*T*_max_	3.58 ± 0.90	3.42 ± 0.51	83.29–116.71%	86.28–113.72%

## Discussion

The analytical method showed good specificity, sensitivity, linearity, precision, and accuracy for the quantitation of OTC in plasma samples, thus allowing its use in bioequivalence assays.

The average plasma profile obtained for the reference (Terramycin LA, Pfizer) and test (Cyamicin LA, Fort Dodge) products were similar, as well as the pharmacokinetic parameters.

The area under the concentration-versus-time curve (AUC) is the parameter that indicates the exposure to the drug in base to the fraction of the dose reaching the systemic circulation and systemic clearance of the drug. In our study, the area under the OTC curve extrapolate to infinity (AUC_0–∞_) was 311.40 ± 93.05 and 287.71 ± 45.31 μg h/mL for R and T formulation, respectively. These values were high; this makes sense due to the prolonged elimination slope. While AUC_0–24_ values were smaller (118.29 ± 45.46 and 131.15 ± 44.56 μg h/mL respectively) compared to those reported by Craigmill et al. (15) in calves receiving the same dose of OTC LA (168 ± 14.6 μg h/mL), and with the area under the curve calculated by Achenbach (16) which was 256.73 μg h/mL, both authors performed these assays on sera samples, while our study was conducted using plasma.

To determine if two formulations would yield the same efficacy behavior, their areas under the respective curves, their maximum concentrations, and the times at which the *C*_max_ is reached, should be determined and compared statistically. If the differences among such parameters are not significant, the drugs are bioequivalent, but if the differences are significant, the formulations are bioinequivalent. In the present work, these parameters were compared through ANOVA, since the populations were normal, using untransformed and log-transformed values. No significant differences were encountered for areas under the curves, and times to the maximum concentrations for the values found were inside the confidence intervals set by the European Union to determine bioequivalence. However, the maximum concentration (*C*_max_) of the test product (Cyamicin LA) was significantly higher than that of the reference formulation and exceeded the bioequivalence interval. These significant differences were found in the untransformed and the log-transformed methodologies and these values were outside the confidence intervals.

The results obtained with i.m. administration of OTC LA in this study for *C*_max_ were similar to those of Ozdemir and Yildirim (17) who reported that *C*_max_ was 8.02 μg/mL at 2.75 h. Although this was a study of the bioequivalence of two LA OTC formulations in sheep, the authors obtained similar results. They mentioned that the multivariate analysis, accomplished through ANOVA for pharmacokinetic parameters (after log-transformation of the data), showed no statistically significant differences between the two products for the parameters *C*_max_ and AUC except between two periods and two groups for AUC. By contrast, much lower *C*_max_ values of 4, 3.89 ± 1.48, and 5.7 ± 0.32 were reported in cattle by Toutain and Raynaud (6), Mestorino et al. (5), and Kumar and Malik (18).

As a final conclusion, the formulations behaved similarly but were not bioequivalent, although *T*_max_ and AUC fell within the bioequivalence criterion. In the case of *C*_max_, a parameter classically considered of importance regarding antimicrobial efficacy, the value obtained after administration of Cyamicin was statistically significantly higher. The 90% confidence interval of T/R was 65.04–134.97% for log-transformation *C*_max_. This interval is not included in the equivalence interval (80–125%), therefore, cannot be considered bioequivalent. The test product showed an extent of absorption appreciably larger than the reference product following administration of the same dose; therefore, suprabioavailability was found. Probably equivalent AUC and *C*_max_ are achieved following administration of a lower dose of the test product as compared to the reference formulation. So could be expected that the two products will have similar systemic efficacy and safety although administered at different doses, but a new bioequivalence assay must be performed.

## Author Contributions

Substantial contributions to the design of the work: NM. Conduction of the field work: animal management and blood sampling: MM and ML (Arg. team). HPLC assay: NM, MM, and ML (Arg. team). Acquisition and analysis of data for the work: NM, MM, and ML (Arg. team). Interpretation of data: NM, PM, IS, CL, and EM. Wrote or contributed to the writing of the manuscript: NM, MM, ML, PM, PZ, CL, IS, and EM. Revised the manuscript: NM, PM, PZ, CL, IS, and EM. The manuscript was critically reviewed and the final version approved by all authors.

## Conflict of Interest Statement

None of the authors of this paper have a financial or personal relationship with other people or organizations that could inappropriately influence or bias the content of the paper.
